# The Braid Index of Complicated DNA Polyhedral Links

**DOI:** 10.1371/journal.pone.0048968

**Published:** 2012-11-20

**Authors:** Xiao-Sheng Cheng, Xian'an Jin

**Affiliations:** 1 Department of Mathematics, Huizhou University, Huizhou, Guangdong, P.R. China; 2 School of Mathematical Sciences, Xiamen University, Xiamen, Fujian, P.R. China; Dalhousie University, Canada

## Abstract

The goal of this paper is to determine the braid index of two types of complicated DNA polyhedral links introduced by chemists and biologists in recent years. We shall study it in a more broad context and actually consider so-called Jaeger's links (more general Traldi's links) which contain, as special cases, both four types of simple polyhedral links whose braid indexes have been determined and the above two types of complicated DNA polyhedral links. Denote by 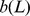 and 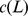 the braid index and crossing number of an oriented link 
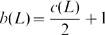
, respectively. Roughly speaking, in this paper, we prove that 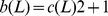 for any link 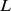 in a family including Jaeger's links and contained in Traldi's links, which is obtained by combining the MFW inequality and an Ohyama's result on upper bound of the braid index. Our result may be used to to characterize and analyze the structure and complexity of DNA polyhedra and entanglement in biopolymers.

## Introduction

The braid index of links has some applications in chemistry and molecular biology. For example, representing knotted hydrocarbon complexes as closed braids [1] can facilitate the study of their properties. In recent 20 years, many DNA polyhedral links such as DNA cube [2], DNA tetrahedron [3], DNA octahedron [4], DNA truncated octahedron [5], DNA bipyramid [6], DNA dodecahedron [7], DNA icosahedron and buckyballs [8] etc, have been synthesized in laboratories or schemes for synthesizing them have been designed by chemists and biologists. In addition, several novel types of polyhedral links have also been posed by mathematicians as a potential object to be synthesized. For four types of simple polyhedral links, their braid indexes have been determined in [9]. The purpose of this paper is to determine braid index of another two types of more complicated polyhedral links appeared in [8], [10], [11], [12], [13] and [14], respectively. We shall study it in a more broad context and actually consider so-called Jaeger's links and more general Traldi's links.

**Figure 1:**
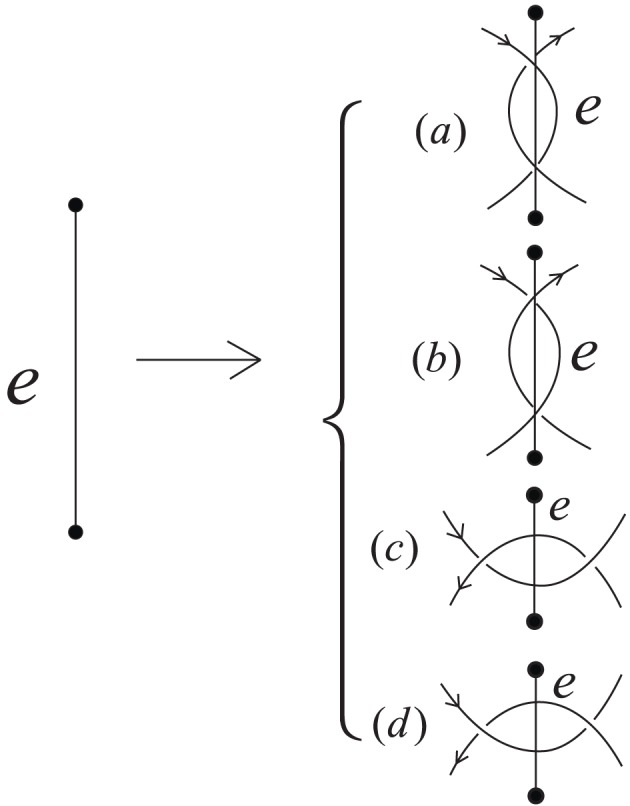
Replace an edge by four types of oriented clasp.

In [15], Jaeger associated to a plane graph an oriented link diagram by replacing each edge of the graph by an oriented clasp as illustrated in 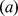 of Figure 1. Then he established a relation between the Tutte polynomial [16] of the graph and the HOMFLY polynomial [17], [18] of the associated oriented link. Moreover, about one year later, both the construction and the relation were extended by Traldi in [19], Traldi constructed his oriented link diagram based on a plane graph via replacing each edge by one of four types of oriented clasps 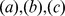 and 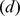, as illustrated in Figure 1. By assigning four different weights to edges of the plane graph according to the four types of oriented clasps he built a relation between the weighted dichromatic polynomial [19] of the graph and the HOMFLY polynomial of the associated oriented link.

Formally, let 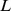 be an oriented link, we call 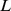 to be a Jaeger's link if 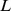, or its reverse, or its mirror image, or its inverse (i.e. the composition of the reverse and the mirror image), has an oriented diagram which can be obtained from a connected plane graph by replacing each edge of the graph by an oriented clasp 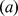 in Figure 1. According to the above definition, Jaeger's links will be all alternating. Analogously, we call 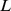 to be a Traldi's link if 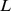, or its reverse, has an oriented diagram which can be obtained from a connected plane graph by replacing each edge of the graph by one of four types of oriented clasps in Figure 1. Note that different edges may be replaced by different types of oriented clasps. Traldi's links may not be alternating, say, the link 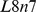 in the Thistlethwaite Link Table [20]. Clearly, Traldi's links contain Jaeger's links as special cases.

The braid index of an oriented link is the minimum number of strings among all closed braid representatives for the given oriented link. We point out that the braid index depends on orientations of links. See Page 215 of [21]. Let 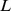 be an oriented link. We denote by 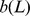 the braid index of 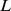. It is, in general, very hard to determine this geometric and numerical invariant 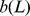 [21]. Let 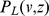 be the HOMFLY polynomial of the oriented link 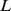. In [22] and [23], Franks, Williams and Morton gave independently a lower bound for the braid index 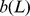 of an oriented link 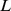 in terms of 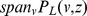 as follows:
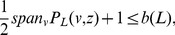
(1)where 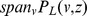 = 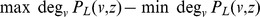, and 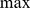 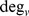 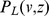 and 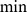 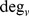 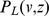 denote, respectively, the maximal degree and minimal degree of 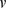 in the polynomial 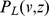. This inequality 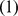 is usually called MFW inequality. The MFW inequality was the first known result relating the braid index to the HOMFLY polynomial. To date, we have known that the MFW inequality for many link families is sharp. For example, the inequality is sharp for all torus links, closed positive n-braids with a full twist [22], 2-bridge links [24], alternating fibred links [24] and a certain family of closed positive braids [25]. In this paper we shall prove the sharpness of the MFW inequality for a family of links including Jaeger's links and contained in Traldi's links. We shall make use of the following result obtained by Ohyama in 1993 [26] which states that for a non-splittable oriented link 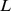,
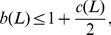
(2)where 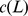 is the crossing number of 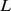. In fact, you will see this upper bound and the MFW lower bound coincide for a family of links including Jaeger's links, but do not coincide for general Traldi's links.

Note that many types of polyhedral links are, in fact, Jaeger's links defined above. Four types of simple polyhedral links considered in [9] are actually all Jaeger's links constructed from subdivisions of (convex) polyhedral graphs or their duals. Note that the dual of a polyhedral graph is also a polyhedral graph and Steinitz's theorem states that a polyhedral graph is a 3-connected simple (i.e. no loops and no multiple edges) planar graph. Thus the result in [9] can not be used to deal with general Jaeger's links. Roughly speaking, in this paper we prove that 
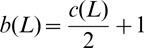
 for any link 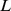 in a family including Jaeger's links and contained in Traldi's links and thus extend the result in [9]. This extension enables us to determine the braid index of another two types of more complicated polyhedral links in [8], [10] and [14] as well as four types of polyhedral links in [9]. It is probable that our result be used to determine braid indexes of new types of polyhedral links to be synthesized in the future. Our research demonstrates that using the braid index or crossing index to describe the complexity of some polyhedral links are equivalent and it may open a door to characterize, analyze the structure and complexity of DNA polyhedra and entanglement in biopolymers.

## Analysis

Before analyzing the braid index of polyhedral links, we give some preliminary knowledge on links, the HOMFLY polynomial, graphs and the dichromatic polynomial.

### 1. Links

A **knot** is a simple closed curve in 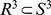, i.e. the image of the embedding of 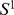 into 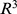. A **link** 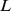 of 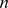 components is a disjoint union of 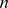 knots: 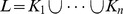. A knot is a link with one component. An oriented link is a link with each of its component assigned an orientation. A **link diagram** is a (regular) projection of a link onto a plane with the under-strand specified by using gaps at each crossing. When the link is oriented, the link diagram will inherit the orientation of the link in a natural way and called oriented link diagram. The **crossing number** or **crossing index** of a link 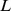, denoted by 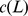, is the least number of crossings that occur in any diagram of the link. A diagram with fewest number of crossings for a given link is called a **minimal** diagram for the link.

**Figure 2:**
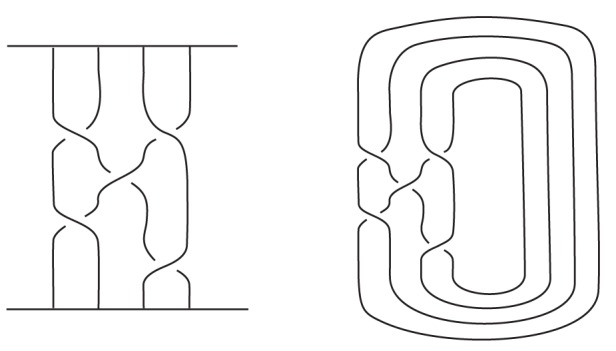
A braid 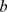 and its corresponding closed braid 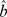.

An 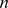 **-string braid** 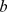 is a set of 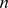 strings in a 3-dimensional cube 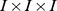, where 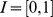, all of which are attached to a horizontal bar at the top 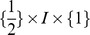 and at the bottom 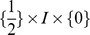 such that each 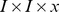, 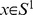, meets the 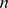 strings in exactly 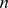 points. A **closed** 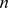 **-string braid** 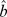 is a set of 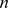 strings embedded in 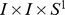 such that each 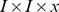, 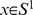, meets the 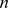 strings in exactly 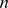 points. An example is illustrated in Figure 2.

It is clear that a closed 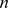-string braid 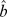 is an oriented link with the convention that we always orient 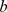 from the top to the bottom. Conversely, Alexander [27], in 1923, showed that every oriented link in 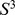 can be represented as a closed 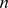-string braid. The **braid index** 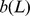 for an oriented link 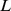 is the smallest positive integer 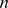 such that 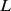 can be represented as a closed 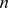-string braid.

Let 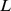 be an oriented link. We denote by 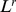 and 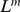 the reverse (reversing the orientation of each component of 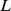) and the mirror image of 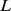, respectively. Then it is obvious that 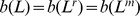.

### 2. The HOMFLY polynomial

**Figure 3:**
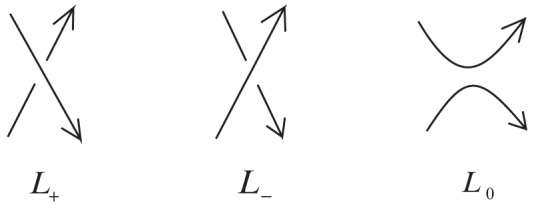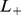, 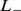 and 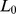.

The HOMFLY polynomial is an invariant of oriented links, introduced in [17] and [18] independently. Now we recall the definition of the HOMFLY polynomial. The HOMFLY polynomial of an oriented link 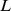, denoted by 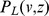, can be defined by the three following axioms [28].

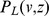
 is invariant under ambient isotopy of L.
2. If 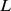 is the trivial knot then 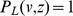.
3. It satisfies the skein relation: 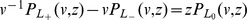, where 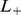, 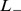 and 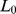 are link diagrams which are identical except near one crossing where they are as in Figure 3 and are called a skein triple.

It possesses the following basic properties [28], which imply that 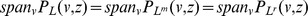.

- If 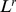 is the reverse of 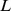, then 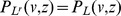.
- If 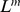 is the mirror image of 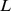, then 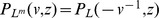.

### 3. Graphs

Let 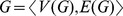 be a graph. We denote by 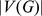 and 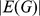 the numbers of vertices and edges, respectively, of the graph 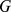. A graph 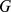 is said to be **connected** if it has a path with two distinct vertices 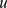 and 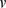 whenever 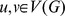 and 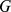 is said to be disconnected otherwise. A **component** of a graph 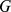 is a subgraph that is connected and is not properly contained in any other connected subgraph of 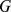. We denote by 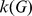 the number of components of the graph 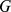. An edge 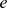 of 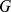 is said to be a **bridge** if 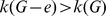. If 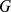 has no bridges, 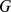 is said to be bridgeless. An edge 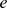 of 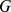 is said to be a **loop** if its two end-vertices are the same. Iff 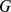 has no loops, 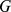 is said to be loopless. The **rank** 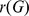 and **nullity** 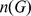 of the graph 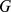 is defined to be 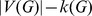 and 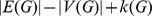, respectively. It is well known that 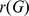 and 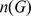 are exactly the dimensions of cycle space and cut space, respectively, of the graph 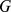 and hence, are both non-negative integers. See, for example, [29].

Given a graph 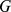 and an edge 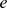 of 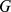, we write 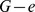 for the graph obtained from 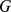 by deleting the edge 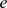, and 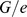 for the graph obtained from 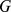 by contracting 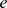, i.e. deleting 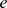 firstly and then identifying its two endvertices. A **planar** graph is a graph which can be embedded in the plane or equivalently, the sphere 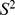. A specific embedding of a planar graph is called a **plane graph**. For undefined notations and terminologies on graph theory, we refer the readers to [29].

### 4. The dichromatic polynomial

A weighted graph is a graph 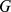 together with a function 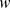 mapping 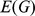 into some commutative ring 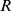 with unity 1. If 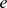 is an edge of the weighted graph 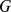, then 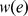 is the weight of the edge 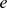. The dichromatic polynomial for weighted graphs was introduced by Traldi in [19], which is a generalization of the Tutte polynomial for signed graphs introduced by Kauffman in [30]. We point out there are actually several weighted versions of the Tutte polynomial.

The dichromatic polynomial 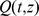 of a weighted graph G can be defined as
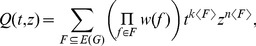
(3)where the summation is over all edge subsets, 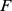, of 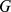, 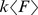 and 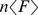 are the number of components and the nullity, respectively, of the spanning subgraph 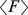, induced by 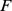, of 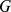. It can also be defined by using the following recursive relations:

1. If 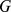 is an edgeless graph with 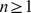 vertices 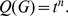

2. If 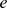 is a loop of 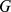, then 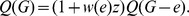

3. If 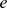 is not a loop of 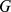, then 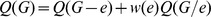.

Just as the Tutte polynomial, it also has a spanning tree expansion and we do not explain it here.

Now we start to analyze the braid index of polyhedral links. We first consider a family of Jaeger's links.

Let 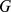 be a connected plane graph. Let 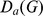 be the oriented link diagram constructed based on 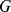 by covering each edge of 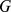 with oriented clasp 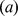 in Figure 1. Oriented link diagrams 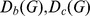 and 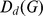 can be defined similarly. Let 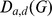 be the oriented link diagram constructed based on 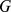 by covering some edges of 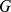 with oriented clasp 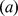 and other edges with oriented clasp 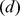 in Figure 1. Oriented link diagrams 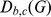 can be defined similarly. Note that 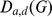 (resp. 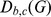) contains 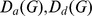 (resp. 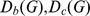) as special cases. More generally, 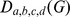 is defined to be the oriented link diagram obtained from 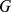 by covering each edge with one of four types of clasps 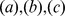 and 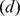. In [19], Traldi obtained the following general formula.

#### Theorem 1

*Let* 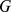 *be a plane graph. Let* 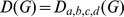 *be the Traldi's oriented link diagram. Let the weight of the edge replaced by oriented clasp* 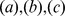 *and* 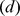 *be* 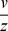, 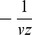, 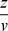 *and* 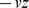, *respectively. Then*

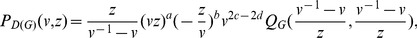
(4)where 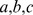 and 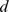 are numbers of edges of 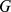 which are replaced by oriented clasp 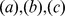 and 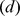, respectively.

The following theorem is a main theoretical tool for determining the braid index of polyhedral links.

#### Theorem 2

*Let* 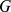 *be a connected bridgeless and loopless plane graph. Let* 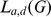 *(resp.* 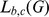 *) be the oriented link that* 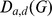 *(resp.* 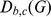 *) represents. Then*

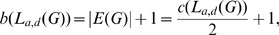
(5)

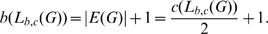
(6)


#### Proof

Note that 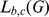 is the mirror image of some 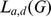 and taking the mirror image does not change the braid index and the crossing index. It suffices for us to prove Eq. (5). When 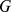 is bridgeless and loopless, it is not hard to observe that 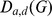 is a connected alternating oriented link diagram without nugatory crossings. According to the famous result [31], [32], [33] due to Kauffman, Murasugi and Thistlethwaite: A connected alternating link diagram without nugatory crossings is minimal, we obtain that
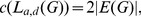
(7)where 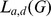 is the link that the link diagram 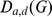 represents. In addition, by a result [34] due to Menasco that states that a link with an alternating diagram will be non-splittable if and only if the diagram is connected, we know that 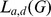 is non-splittable. Thus, by Eq. (2), we have
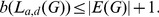
(8)


Now we consider the lower bound of 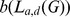. For the oriented link 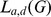, as a special case of Theorem 1, we obtain
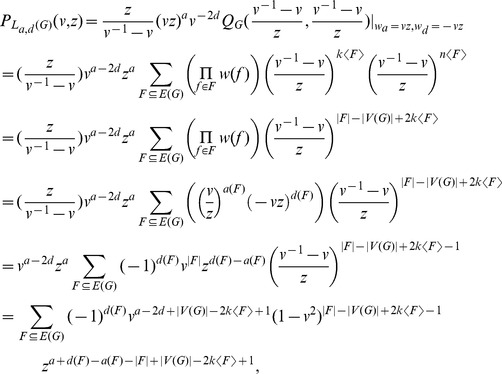
(9)


where 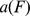 and 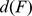 are numbers of edges in 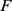 covered by clasps of types 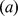 and 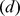, respectively. It is clear that 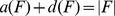.

Now we compute the highest and lowest degrees in 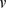 of Eq. (9). Note that the power of 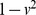 is 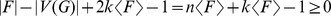. Thus we have
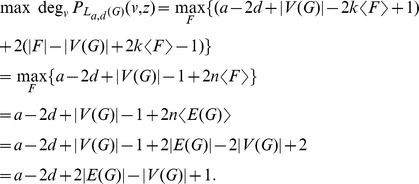
The third equation holds since 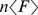 attains maximality if and only if 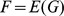 because of 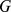 is bridgeless. Similarly, we have
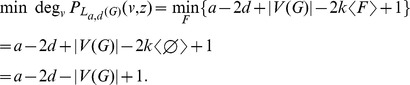
The second equation holds since 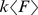 attains maximality if and only if 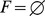 since 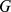 is loopless. Thus,
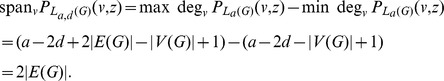
Hence, by Eq. (1), we obtain
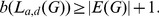
(10)


Combining Eq. (8) with Eq. (10), we show that Eq. (5) holds. 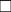


#### Corollary

*(1). Let* 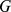 *be a connected bridgeless plane graph. Then*

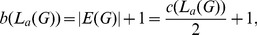
(11)

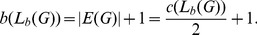
(12)
*(2). Let* 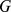 *be a connected loopless plane graph. Then*

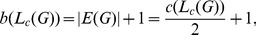
(13)

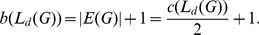
(14)
*As a result,*

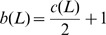
(15)for any Jaeger's link 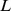.

#### Proof

**Figure 4:**
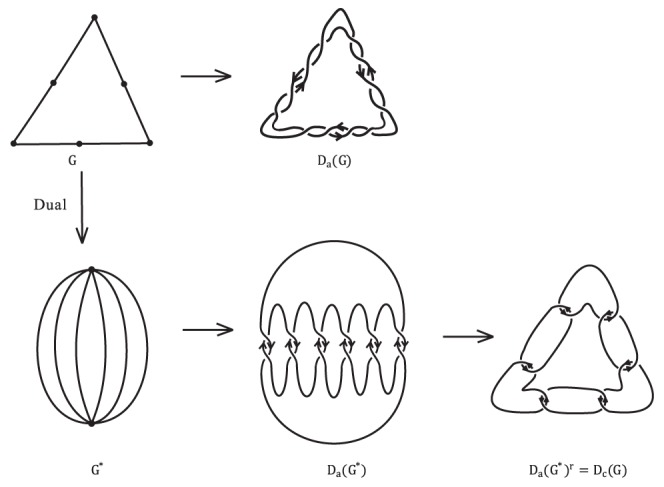
An example.


By checking the proof of Theorem 2, we find that the condition “loopless” is actually not necessary for the special case 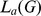. Hence, Eq. (11) holds. Eq. (12) follows from Eq. (11) and the fact 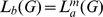. Let 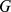 be a connected loopless plane graph and 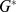 be the planar dual of 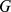. Then 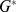 is connected and bridgeless. Note that 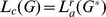 (see Figure 4 for an example) and 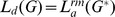 and reversing orientations do not change braid index and crossing index. Hence, Eqs. (13) and (14) hold. By the definition of a Jaeger's link 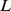, we know that there is a connected plane graph 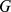 such that 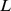 is equivalent to one of the four links: 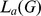, 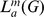, 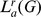 and 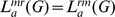. It will suffice to prove that 
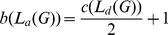
 for any connected plane graph 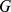. Firstly, 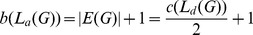 for any connected bridgeless plane graph 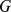. Secondly, if 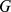 has bridges, let 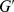 be the connected plane graph obtained from 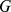 by contracting all bridges of 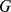. Note that 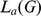 is equivalent to 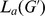 via untwisting oriented clasps covering bridges. Hence, we have 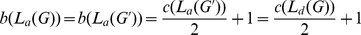.

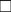


#### Remark

*Eq. (15)* *generalizes Theorem 4.3 in* [*9*] *from subdivisions of polyhedral graphs to any connected plane graphs.*

**Figure 5:**
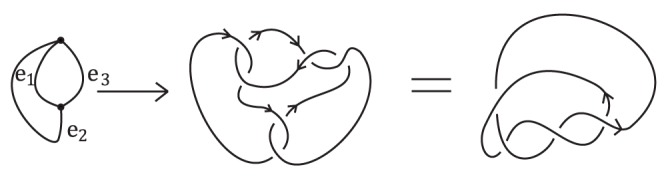
An alternating Traldi's link upper and lower bounds of whose braid index are not equal.

To conclude the section, we point out that the braid index of general Traldi's links (even for alternating Traldi's links) can not be determined similarly since the upper and lower bounds are not always equal in general. An example of alternating Traldi's links, denoted by 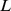, is shown in Figure 5 which is constructed from a theta graph by replacing edges 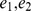 by oriented clasp 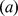 and 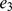 by clasp 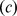. On the one hand, by using the software KnotGTK, we obtain
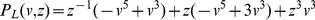
(16)and hence, 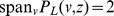. On the other hand, we have 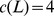. Therefore,
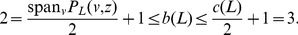
(17)


## Results

It is obvious that by the Corollary the braid index of four types of polyhedral links (see Fig. 2 of [9]) can be determined. In this section, we determine the braid index of two types of complicated DNA polyhedral links. However, braid indexes of these two new types of polyhedral links can not be obtained by the previous result in [9]. You will see these new types of polyhedral links are all alternating and hence their crossing numbers are easily obtained. By the Corollary, in order to obtain the braid index, we only need to show that these links are Jaeger's links, and equivalently to find their associated plane graphs. Note that two strands of DNA have antiparallel orientations which are consistent with Jaeger's orientations. For simplicity, we do not draw orientations in the following any more.

### 1. Double crossover DNA polyhedral links

**Figure 6:**
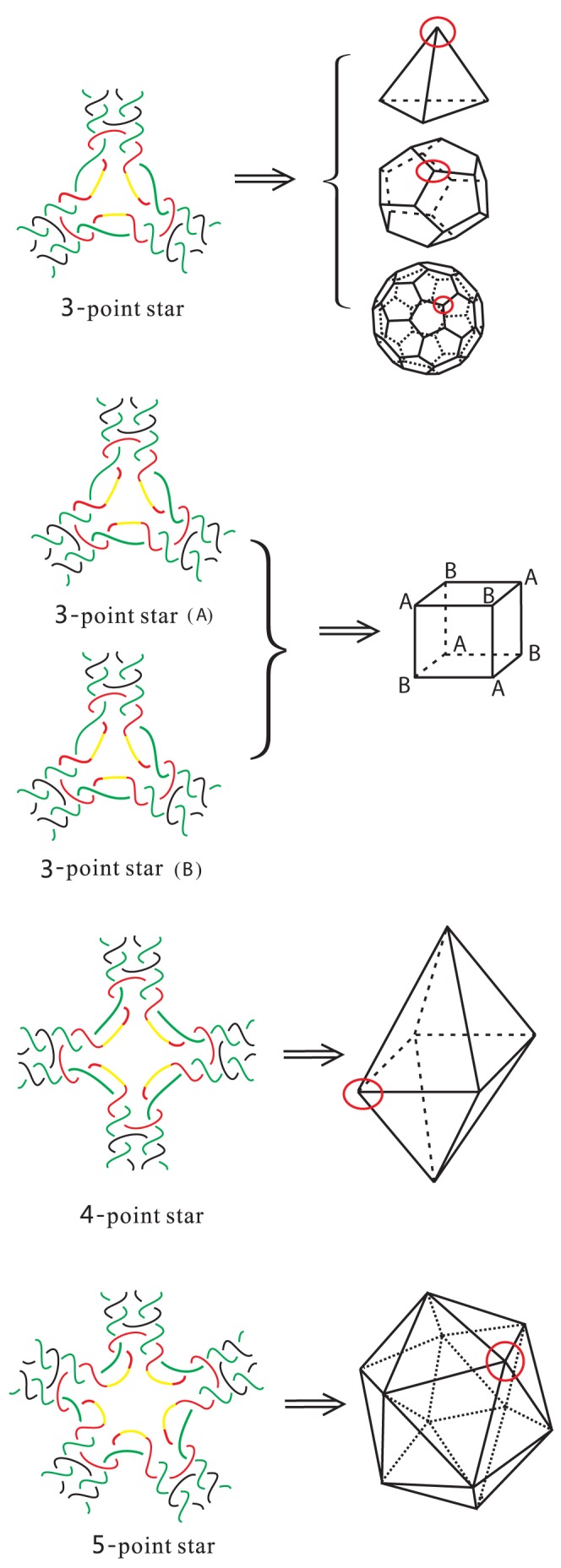
3-point star motifs: DNA tetrahedron, cube, dodecahedron, buckyball; 4-point star motifs: DNA octahedron; 5-point star motifs: DNA icosahedron.

Recent years, in [8], [10], [11], [12], [13], the authors, in laboratory, designed and synthesized some fancy double crossover DNA polyhedra, such as DNA tetrahedron, cube, octahedron, dodecahedron, icosahedron and buckyball, by covering each vertex of degree 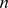 of the polyhedron by “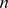-point star motif (tiles)” and through sticky-end association between the tiles. The “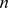-point star motif” has an 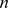-fold rotational symmetry and consists of 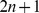 single strands: a long repetitive central DNA strand (colored red and yellow), 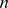 identical medium DNA strands (colored green) and 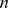 identical short DNA strands (colored black). The colored yellow part at the center of the motif represents 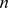 unpaired DNA single-strands whose lengths can be adjusted to change bending degree of the whole structure. See Figure 6.

We point out in Figure 6 the tiles are a little different in the ends of tiles from the actual assemblies of such DNA polyhedra. For actual assemblies, we refer the reader to Fig. 1 of [8] for DNA tetrahedron, dodecahedron and buckyball, Fig. 4 of [11] for DNA cube, Fig. 1 of [13] for DNA octahedron and Fig. 4(f) of [10] and Fig. 1 of [12] for DNA icosahedron. However, one will obtain the same polyhedron links based on our drawings as actual assemblies, and hence do not affect the analysis of their braid indexes.

**Figure 7:**
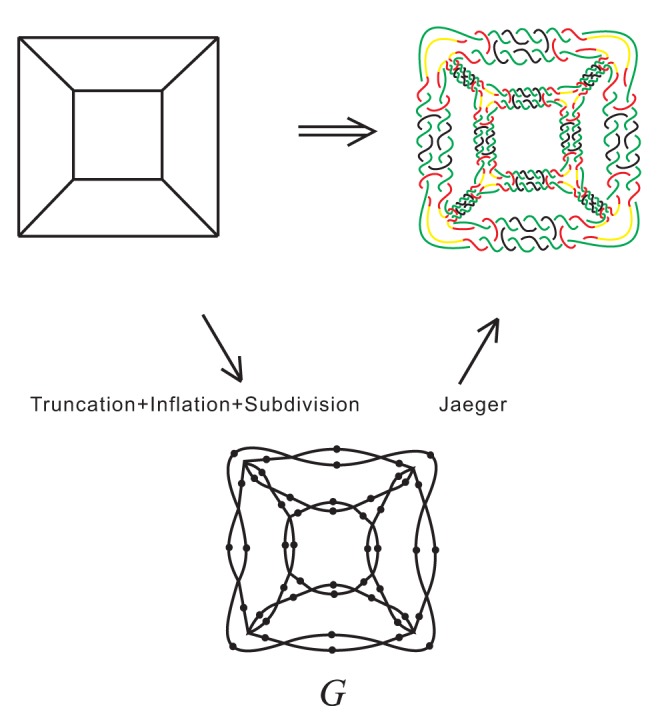
Planar form of double crossover DNA cube and its associated plane graph 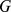.

Now we take double crossover DNA cube as an example to show double crossover DNA polyhedra are all Jaeger's links. The planar form of double crossover DNA cube link and its associated plane graph are shown in Figure 7 (upper and right) and (lower), respectively. In general, the associated plane graph 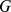 can be obtained from the polyhedron 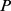 by the following steps:

**Step 1:** Truncating the polyhedron 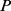, i.e. cutting each corner of the polynhedron 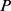, we obtain a polyhedral graph 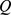.

**Step 2:** Inflating edges of 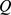 corresponding to edges of 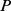, i.e. converting a single edge into two parallel edges, we obtain a plane graph 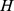.

**Step 3:** Subdividing each edge of 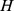 by inserting a vertex, we obtain the associated graph 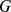.

Let 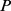 be a polyhedron with 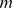 edges. Then, by simple calculation, the associated plane graph 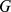 has 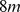 edges and thus the braid index of the corresponding double crossover DNA polyhedral link is 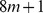.

### 2. Cycle-crossover polyhedral links

**Figure 8:**
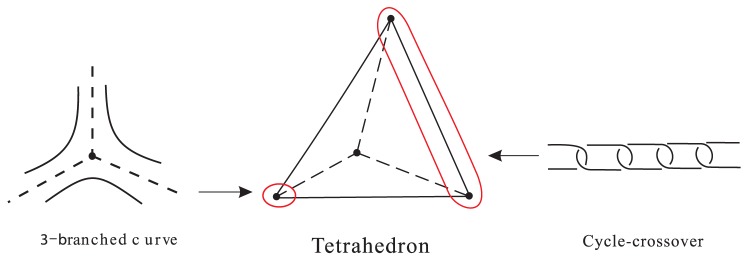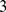-branched curve, cycle-crossover and the construction of cycle-crossover tetrahedral link.

**Figure 9:**
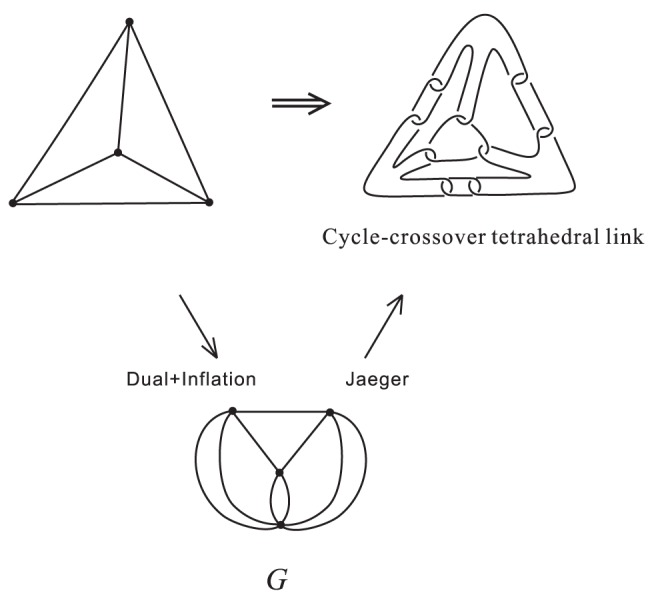
Planar form of cycle-crossover DNA tetrahedron and its associated plane graph 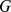.

This type of polyhedral links was introduced in [14], designed as a potential object for synthesising, which can be constructed from a polyhedron by replacing each edge of the polyhedron by a “cycle-crossover” and replacing each vertex of degree 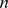 by an 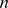-branched curve. See Figure 8. The planar form of the cycle-crossover cube link is shown in Figure 9 (upper and right) and its associated plane graph is shown in Figure 9 (lower).

In general, cycle-crossover polyhedral links are all Jaeger's links. The associated plane graph 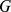 can be obtained from the polyhedron 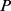 by the following steps:

**Step 1:** Computing the planar dual of 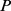, we obtain a plane graph 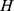


**Step 2:** Inflating edges of 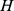, i.e. converting a single edge into several parallel edges, we obtain the associated graph 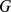.

In this case suppose that the total lengths of cycle-crossovers used to construct cycle-crossover polyhedral links are 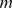. Then the associated plane graph 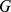 has 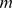 edges and thus the braid index of the corresponding cyle-crossover DNA polyhedral link is 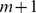.

## Discussion

Firstly, note that the two associated plane graphs in Figures 7 and 9 are both not subdivisions of polyhedral graphs and for both types of polyhedral links. This means that the method used in [9] can not be used here. Secondly, it is easy to find that the braid index of two types of complicated polyhedral links does not depend on the structure of the polyhedron. Thirdly, as the referee points out that there are two types of DNA cube: 4 turns and 4.5 turns. See Figure 4 and Figure 1, respectively, of [11], we only consider the 4-turn DNA cube in this paper. It deserves to study the braid index of 5-turn DNA cube and more general 5-turn DNA polyhedra. Finally, in fact Theorem 2 is more powerful than it has been used, and hence may be used to determine the braid index of more complicated polyhedral links in the future.

## References

[1] Cox MA, Hughes TS, Ellis-Monaghan JA, Mondanaro KR (2008) Hydrocarbon links in an octet truss. J Math Chem 43: 874–891.

[2] Chen J, Seeman NC (1991) Synthesis from DNA of a molecule with the connectivity of a cube. Nature 350: 631–633. 2017259 10.1038/350631a0

[3] Goodman RP, Berry RM, Turberfield AJ (2004) The single-step synthesis of a DNA tetrahedron. Chem Commun 12: 1372–1373. 10.1039/b402293a 15179470

[4] Shih WM, Quispe JD, Joyce GF (2004) A 1.7-kilobase single-stranded DNA that folds into a nanoscale octahedron. Nature 427: 618–621. 14961116 10.1038/nature02307

[5] Zhang Y, Seeman NC (1994) The construction of a DNA truncated octahedron. J Am Chem Soc 116: 1661–1669.

[6] Erben CM, Goodman RP, Turberfield AJ (2007) A Self-Assembled DNA Bipyramid. J Am Chem Soc 129: 6992–6993. 17500526 10.1021/ja071493b

[7] Zimmermann J, Cebulla MPJ, Mönninghoff S, Kiedrowski GV (2008) Self-Assembly of a DNA Dodecahedron from 20 Trisoligonucleotides with C3h Linkers. Angew Chem Int Ed 47: 3626–3630. 10.1002/anie.200702682 18383496

[8] He Y, Ye T, Su M, Zhang C, Ribbe AE, et al (2008) Hierarchical self-assembly of DNA into symmetric supramolecular polyhedra. Nature 452: 198–202. 18337818 10.1038/nature06597

[9] Cheng XS, Jiang X, Dai H (2012) The braid index of polyhedral links. J Math Chem 50: 1386–1397.

[10] Lin C, Liu Y, Yan H (2009) Designer DNA Nanoarchitectures. Biochemistry 48 (8) 1663–1674. 19199428 10.1021/bi802324w PMC2765550

[11] Zhang C, Ko SH, Su M, Leng Y, Ribbe AE, et al (2009) Symmetry Controls the Face Geometry of DNA Polyhedra. J Am Chem Soc 131: 1413–1415. 19173666 10.1021/ja809666h

[12] Zhang C, Su M, He Y, Zhao X, Fang P, et al (2008) Conformational flexibility facilitates selfassembly of complex DNA nanostructures. Proc Natl Acad Sci USA 105: 10665–10669. 18667705 10.1073/pnas.0803841105 PMC2504817

[13] He Y, Su M, Fang P, Zhang C, Ribbe AE, et al (2010) On the chirality of self-assembled DNA octahedra. Angew Chem Int Ed 49: 748–751. 10.1002/anie.200904513 20017168

[14] Cheng XS, Zhang HP, Hu G, Qiu WY (2010) The architecture and Jones polynomials of cycle-crossover polyhedral links. MATCH Commun Math Comput Chem 63: 637–635.

[15] Jaeger F (1988) On Tutte polynomials and link polynomials. Proc Amer Math Soc 103: 647–654.

[16] Tutte WT (1954) A contribution to the theory of chromatic polynomials. Canad J Math 6: 80–91.

[17] Freyd P, Yetter D, Hoste J, Lickorish WBR, Millett K, et al (1985) A new polynomial invariant of knots and links. Bull Amer Math Soc (NS) 12: 239–246.

[18] Przytycki JH, Traczyk P (1987) Invariants of links of Conway type. Kobe J Math 4: 115–139.

[19] Traldi L (1989) A dichromatic polynomial for weighted graphs and link polynomials. Proc Amer Math Soc 106: 279–286.

[20] Math.toronto.edu. Available: http://katlas.math.toronto.edu/wiki/The Thistlethwaite Link Table. Accessed 2012 Oct 11.

[21] Murasugi K (1996) Knot theory and its applications. Boston: Birkhauser. 341 p.

[22] Franks J, Williams RF (1987) Braids and the Jones polynomial. Trans Amer Math Soc 303: 97–108.

[23] Morton HR (1986) Seifert circles and knot polynomials. Math. Proc. Cambridge Philos Soc 99: 107–109.

[24] Murasugi K (1991) On the braid index of alternating links. Trans Amer Math Soc 326: 237–260.

[25] Nakamura T (2004) Notes on the braid index of closed positive braids. Topology Appl 135: 13–31.

[26] Ohyama Y (1993) On the minimal crossing number and the braid index of links. Canad J Math 45: 117–131.

[27] Alexander JW (1923) A lemma on systems of knotted curves. Proc Natl Acad Sci USA 9: 93–95. 16576674 10.1073/pnas.9.3.93 PMC1085274

[28] Cromwell PG (2004) Knots and Links. Cambridge University Press. 328 p.

[29] Bondy JA, Murty USR (1976) Graph theory with applications. London and Basingstoke: The Macmillan Press Ltd. 264 p.

[30] Kauffman LH (1989) A Tutte polynomial for signed graphs. Discrete Appl Math 25: 105–127.

[31] Kauffman LH (1987) State models and the Jones polynomial. Topology 26: 395–407.

[32] Thistlethwaite MB (1987) A spanning tree expansion of the Jones polynomial. Topology 26: 297–309.

[33] Murasugi K (1987) Jones polynomial and classical conjectures in knot theory. Topology 26: 187–194.

[34] Menasco W (1984) Closed incompressible surfaces in alternating knot and link complements. Topology 23 (1) 37–44.

